# Is a giant incisional hernia a contraindication for laparoscopic cholecystectomy?

**DOI:** 10.1093/jscr/rjad305

**Published:** 2023-06-17

**Authors:** Nicolas Boyer, Nikolaos Koliakos, Luca Pau, Mathilde Poras, Marie-Therese Maréchal, Eleonora Farinella

**Affiliations:** Department of Digestive Surgery, Saint-Pierre University Hospital, Université Libre de Bruxelles, Brussels, Belgium; Department of Digestive Surgery, Saint-Pierre University Hospital, Université Libre de Bruxelles, Brussels, Belgium; Department of Digestive Surgery, Saint-Pierre University Hospital, Université Libre de Bruxelles, Brussels, Belgium; Department of Digestive Surgery, Saint-Pierre University Hospital, Université Libre de Bruxelles, Brussels, Belgium; Department of Digestive Surgery, Saint-Pierre University Hospital, Université Libre de Bruxelles, Brussels, Belgium; Department of Digestive Surgery, Saint-Pierre University Hospital, Université Libre de Bruxelles, Brussels, Belgium

**Keywords:** laparoscopic, cholecystectomy, incisional hernia

## Abstract

Laparoscopic cholecystectomy (LC) is one of the most commonly performed surgical procedures worldwide. A previous abdominal operation is not considered a significant risk factor for conversion to open cholecystectomy. We describe the case of an 80-year-old woman with a surgical history of a giant uncomplicated incisional midline hernia presenting at our department with choledocholithiasis and acute cholangitis. After an ERCP with extraction of common bile duct stones, a LC was planned. The first trocar was inserted in the right midclavicular line, using an open technique and a careful inspection of the abdominal cavity and the hernia sac content. An uncomplicated cholecystectomy was performed and the postoperative course was uneventful.

## INTRODUCTION

Laparoscopic cholecystectomy (LC) has been considered the gold standard operation for complicated and symptomatic cholelithiasis [1]. It has been 30 years since the first published randomised trial mentioned the advantageous postoperative outcomes over the open technique [2]. However, recent studies have revealed that conversion rates to open surgery in the era of LC remain surprisingly high. Several preoperative and intraoperative risk factors for conversion to open surgery have been reported [3, 4]. The active role of adhesions following a previous open abdominal surgery remains disputable, previous surgery is not an absolute predictor of conversion.

We present an 80-year-old woman with a history of a giant postoperative midline hernia successfully treated laparoscopically for complicated cholelithiasis.

## CASE REPORT

An 80-year-old woman was admitted to our department for evaluation of epigastric abdominal pain. Her past medical history included hypertension, aortic valve disease and chronic obstructive pulmonary disease. Her past surgical history included an uncomplicated incisional hernia following an open small bowel resection for ischemia because of volvulus. There was significant loss of domain and clinically there was a distance of 15 cm between the edges of the rectus sheath. The patient was hemodynamically stable and afebrile. Physical examination revealed right upper quadrant tenderness and a large reducible abdominal wall hernia at the site of a previous midline incision (10^*^15 cm). Laboratory data at the time of admission were as follows: white blood cells: 17.450/μL, CRP: 82 mg/dl, total-value bilirubin: 1.8 mg/dL, direct bilirubin: 1.4 mg/dL, aspartate aminotransferase: 205 IU/L, alanine aminotransferase: 153 IU/L, alkaline phosphatase: 523 IU/L and γ-glutamyl transpeptidase: 486 IU/L.

**Figure 1:**
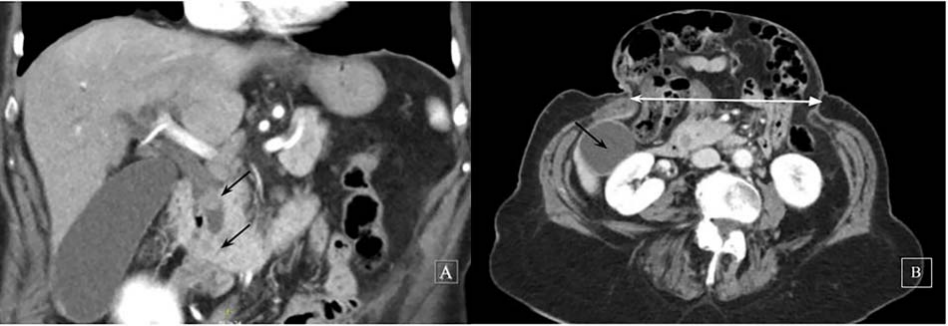
Contrast-enhanced computed tomography: a coronal image revealing two iso-attenuating filling defects in the distal bile duct causing obstruction (black arrows) (**A**) and a subcostal axial slice of a giant incisional hernia (horizontal distance 14 cm, white line, GB, black arrow) (**B**)

Abdominal U/S revealed the dilatation of the intrahepatic and extrahepatic bile ducts (common bile duct [CBD] diameter: 13 mm) and gallbladder (GB) sludge and the presence of a gallstone in the Hartmann’s pouch. Contrast-enhanced abdominal CT confirmed two iso-attenuating filling defects in the distal bile duct causing obstruction (Fig. 1). The CT also helped delineate the hernia as: contents, size and distance between rectus sheaths. ERCP with sphincterotomy and balloon dilatation was eventfully performed. The patient’s clinical course was uneventful and an elective LC was planned. Out of fear for complications the patient refused elective hernia repair.

**Figure 2:**
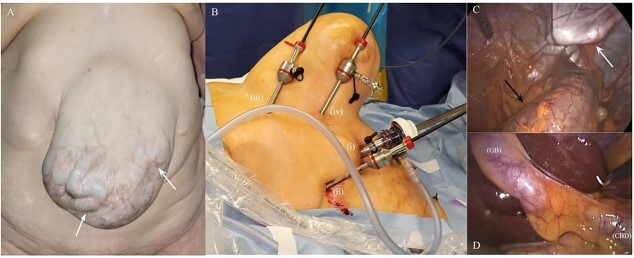
Intraoperative images. Giant incisional hernia with peristaltic movements in a loop of small intestine (white arrows) (**A**); trocar placement in relation to the incisional hernia: first 10-mm trocar in the midclavicular line (i), 5-mm trocar subcostally in the anterior axillary line (ii), 5-mm assistant-port in the epigastrium (iii) and 5-mm working-port parallel to the subcostal plane in tangential position to the right border of the hernia (iv) (**B**); laparoscopic view of the hernia sac pushed against surgeon’s finger (white arrow) and the transverse colon below (black arrow) (**C**); laparoscopic view of the GB and the CBD (**D**)

After preparation of the surgical site (Fig. 2A), the first 10-mm trocar was placed in the right midclavicular line, using an open technique. After insufflation of the abdomen to 12 mmHg and the insertion of a 30-degree laparoscope, one 5-mm trocar was induced 3 cm subcostally in the anterior axillary line (Fig. 2B). Two other 5-mm trocars were placed in tangential position to the right border of the hernia, the first assistant-port in the epigastrium and the second caudally and parallel to the subcostal plane. The hernia sac could be visualised containing the transverse colon without the presence of firm adhesions (Fig. 2C). An uncomplicated cholecystectomy was performed after a safe ligation of cystic duct and artery with polymeric clips (Fig. 2D). The postoperative course was uneventful and the patient was discharged after 24 h.

## DISCUSSION

LC remains one of the most commonly performed surgical procedures worldwide. According to a very recent meta-analysis, early LC remains the recommended intervention even for patients suffering from severe acute cholecystitis (AC) [5]. In contrast, septic shock and admission to the intensive care unit because of AC complications constitute the only relative contra-indications of LC [5].

Whilst laparoscopic adhesiolysis leads to longer operating time, more postoperative complications and increased length of stay [6], previous abdominal surgery is not a significant risk factor for conversion to open cholecystectomy [3]. A systematic review by Rothman et al. [3] showed that AC, GB thickness, advanced age and male gender were independent risk factors for conversion. More specifically in 2019, a multicentre, multinational study showed an overall conversion rate to open surgery of 14.3%, and a rate of 7.5% in elective and 22.4% in emergency cases, respectively [4].

The current report describes a case of an 80-year-old patient, suffering from complicated cholelithiasis requiring LC.. She has denied the repair of her massive ventral hernia because of multiple medical comorbidities. After the uncomplicated completion of the ERCP stones extraction, a LC was decided without a second thought.

Several authors have already used modified trocar positions based on classical four-port technique in patients with previous abdominal operations to avoid surgical scars [7, 8]. Given the clinical presentation with a large midline incisional hernia in this patient, an alternative trocar positioning was used to avoid entering the hernia sac. Inserting the first trocar using the open technique in the right lateral abdominal wall enabled a careful inspection of the abdominal cavity and the hernia sac content. After this, the other trocars could be safely positioned under direct vision avoiding complications. The patient had an uneventful recovery.

In conclusion, LC following previous abdominal surgical procedures is feasible in expert hands. Insertion of the first trocar at an optimal distance from the midline hernia defect leads to optimal visualisation of the hernia sac and aids in positioning other trocars in a modified position safely.

## CONFLICT OF INTEREST STATEMENT

None declared.

## FUNDING

None.

## INFORMED CONSENT

Written informed consent for publication of their clinical details and/or clinical images was obtained from the patient/parent/guardian/relative of the patient.

## DECLARATION OF PATIENT CONSENT

The authors certify that they have obtained all appropriate patient consent forms. In the form, the patient has given his consent for his images and other clinical information to be reported in the journal. The patient understands that name and initials will not be published and due efforts will be made to conceal identity, but anonymity cannot be guaranteed.

## DATA AVAILABILITY

The data that support the findings of this study are available from the corresponding author upon reasonable request.
